# Measurements of Bone Health after Thyroid-Stimulating Suppression Therapy in Postmenopausal Women with Differentiated Thyroid Carcinoma: Bone Mineral Density versus the Trabecular Bone Score

**DOI:** 10.3390/jcm10091964

**Published:** 2021-05-03

**Authors:** Chae Won Chung, Hoon Sung Choi, Sung Hye Kong, Young Joo Park, Do Joon Park, Hwa Young Ahn, Sun Wook Cho

**Affiliations:** 1 Department of Internal Medicine, Seoul National University Hospital, Seoul 03080, Korea; jjwon9523@gmail.com (C.W.C.); yjparkmd@snu.ac.kr (Y.J.P.); djpark@snu.ac.kr (D.J.P.); 2 Department of Internal Medicine, Kangwon National University Hospital, Chuncheon-si 24289, Korea; hoonsung80@gmail.com; 3 Department of Internal Medicine, Seoul National University College of Medicine, Seoul 03080, Korea; shkong@snu.ac.kr; 4 Department of Internal Medicine, Seoul National University Bundang Hospital, Kyonggi 13620, Korea; 5 Department of Internal Medicine, Chung-Ang University Hospital, Seoul 06973, Korea; ahy0809@gmail.com

**Keywords:** differentiated thyroid carcinoma, bone mineral density, trabecular bone score, TSH suppression therapy

## Abstract

Background: Thyroid-stimulating hormone (TSH) suppression therapy is an important treatment modality for differentiated thyroid carcinoma (DTC), but it increases fracture risk. The aim of this study was to evaluate changes in bone mineral density (BMD) and trabecular bone score (TBS) in postmenopausal DTC patients receiving TSH suppression therapy. Methods: A total of 410 postmenopausal DTC patients who underwent thyroidectomy and had at least two dual-energy X-ray absorptiometry measurements, including a preoperative measurement, were included. Patients who had osteoporosis medication for more than 1 year were classified as ‘patients with osteoporosis’. Results: In patients without osteoporosis, the change in %BMD was similar between TSH suppression (−) and (+) groups, while the decrease in %TBS was significantly greater in the TSH suppression (+) group than that of the TSH suppression (−) group. The relative risk of vertebral fracture was decreased by TBS changes but not by BMD changes. In patients with osteoporosis, both BMD and TBS showed significant increases in the TSH suppression (−) group but not in TSH suppression (+) group. At year 4, TBS was significantly lower in the TSH suppression (+) group than that in the TSH suppression (−) group, while BMD showed no difference between groups. Conclusions: TBS may better reflect bone health than BMD in postmenopausal DTC patients with TSH suppression therapy.

## 1. Introduction

Differentiated thyroid carcinoma (DTC) is the most common endocrine cancer, and it has a favorable prognosis with low mortality rate [1,2]. In fact, the thyroid cancer-specific mortality was 1.4% and the recurrence rate was 13.3% during a five-year follow-up in South Korea [3]. Thyroid-stimulating hormone (TSH) suppression therapy is under debate as it was prolonged following the long-term survival of DTC patients. Because the growth of thyroid cancer cells is enhanced by thyroid-stimulating hormone (TSH) through receptors expressed in cancer cells [4], TSH suppression therapy has been used to prevent recurrence of thyroid cancer since 1977 [5,6]. However, considerable frequency of side effects such as atrial fibrillation, thyrotoxicosis, and osteoporosis [7,8,9,10] has been reported.

The main reason for these side effects, especially the decrease in bone density, is persistent subclinical hyperthyroidism induced by TSH suppression therapy [7,11]. This thyrotoxic status causes an imbalance in bone remodeling, induces bone resorption, and eventually decreases bone density [12,13]. Decreased bone density places patients at an elevated risk for fragility fracture, which is particularly problematic in older adults and is associated with a mortality rate of 20–33% at 1 year after fracture [14]. Therefore, given the high long-term survival rate of DTC, bone health monitoring is of paramount importance in DTC patients receiving TSH suppression therapy, especially in postmenopausal women, who are particularly vulnerable to bone loss.

Bone mineral density (BMD) has so far been the major tool used to obtain quantitative information on bone health and to anticipate the risk of fracture. However, increasingly, many studies are questioning the effectiveness of BMD. According to recent studies, 50% of fragility fractures occur in patients with a T-score greater than −2.5, the criterion used to define osteoporosis based on BMD [15,16]. To enhance the prediction of fragility fracture risk, the use of the trabecular bone score (TBS) is increasing [17,18,19]. TBS is an independent parameter calculated by measuring gray-level texture, which is used to estimate the trabecular microstructure, based on two-dimensional dual-energy X-ray absorptiometry (DXA) images [17]. Since it is not affected by other factors that increase BMD, including aortic calcification, osteophytes formation or fractures, TBS can predict fracture risk more accurately than BMD, even in patients with a BMD T-score higher than −2.5 [17].

Limited research has investigated the use of TBS in DTC patients, and the results have been inconsistent [20,21]. Therefore, we analyzed changes in BMD and TBS in postmenopausal DTC patients according to whether TSH suppression therapy was applied.

## 2. Methods

### 2.1. Subjects

Postmenopausal women with DTC who underwent thyroidectomy between January 2008 and December 2017 at Seoul National University Hospital and Chung-Ang University Hospital were retrospectively reviewed. Patients who had a DXA scan within 6 months before the operation date and follow-up DXA scans at 2 and/or 4 years postoperatively were included. The following exclusion criteria were applied: (1) history of medication affecting bone metabolism (e.g., glucocorticoids, warfarin, etc.); (2) recurrence of thyroid cancer or distant metastasis during follow-up; (3) postoperative hypoparathyroidism or hyperparathyroidism; (4) other malignancy within 5 years before surgery; (5) gastrointestinal absorptive disorder; (6) renal or hepatic impairment; and (7) pregnancy. The study was conducted in accordance with the Declaration of Helsinki and was approved by the Institutional Review Board of the Seoul National University Hospital (IRB 1902-075-1011) and the Chung-Ang University Hospital (IRB 1904-007-16258).

All enrolled patients were divided into two groups. Patients who were diagnosed with osteoporosis, initiated osteoporosis medication within 1 year before surgery and who maintained it for more than 1 year were designated as ‘patients with osteoporosis’. Osteoporosis medication included bisphosphonate, denosumab, selective estrogen receptor modulator (SERM), and hormone replacement therapy (HRT). Patients who had osteoporosis but had not received osteoporosis medication for more than 1 year were excluded. Others were designated as ‘patients without osteoporosis’.

The mean TSH level of each patient was defined as the integral value of TSH over time divided by the total follow-up period. Before 2015, patients underwent TSH suppression therapy, targeting TSH under 0.05 mIU/L. if they had any of the following pathologic findings after surgery: (1) tumor size ≥4 cm, (2) LN metastasis, (3) extrathyroidal extension, (4) multiplicity, (5) histologic aggressive variant, or (6) distant metastasis. After 2015, all patients underwent stratified TSH suppression therapy after surgery or readjusted TSH suppression regimen by risk of recurrence as specified in the 2015 American Thyroid Association Management Guideline for adult patients with DTC. Patients with a mean TSH level lower than 1.0 mIU/L were designated as the TSH suppression (+) group. The others were designated as the TSH suppression (−) group.

### 2.2. Biochemical Parameters

Serum samples were obtained from 8 a.m. to 9 a.m. and analyzed within 24 h. Plasma 25-hydroxy (OH) vitamin D levels were measured by the radioimmunoassay technique (DiaSorin Inc., Stillwater, MN, USA), with an interassay coefficient of variation (CV) of 11.1% and an intraassay CV of 8.8%. Concentrations of serum fT4 and TSH were measured by immunoradiometric assays (fT4 (DiaSorin S.p.A.) and TSH (OCPL07-TSH), CIS Bio International, Gif-sur-Yvette, France). The TSH measurements had an analytical sensitivity of 0.04 mIU/L and a functional assay sensitivity of 0.07 mIU/L. The reference ranges for fT4 and TSH were from 0.89 to 1.79 ng/dL and from 0.3 to 4.0 mIU/L, respectively.

### 2.3. Assessment of Vertebral Bone Mineral Density (BMD) and Trabecular Bone Score (TBS)

The BMD (g/cm^2^) of the lumbar spine was measured based on DXA images (GE Lunar Prodigy, GE Healthcare, Madison, WI, USA) using Encore software version 11.0 (GE Healthcare, Chicago, IL, USA), according to the manufacturer’s guidelines. The precision error in BMD of the lumbar spine was 1.7%. Lumbar spines with compression fractures or severe sclerotic changes were excluded during the image acquisition process.

The TBS of the lumbar spine was assessed by TBS iNsight Software, version 2.0.0.1 (Med-Imaps, Pessac, France) using the lumbar spine DXA file.

### 2.4. Assessment of Incidence and Relative Risk of Fracture

The incidence of vertebral fracture was confirmed by Genant classification based on vertebral shape [22]. The relative risk of fracture per 1 standard deviation (SD) change in lumbar spine BMD or TBS over time was calculated by Cox proportional hazards model. The model was adjusted for baseline 10-year major fracture and hip fracture risk estimated by FRAX score assessment for South Korea.

### 2.5. Statistical Analyses

The Student *t*-test was used for between-group comparisons of continuous variables. A linear mixed model was conducted to analyze data with unequal numbers of repetitions. *p*-values < 0.05 were considered to indicate statistical significance. Cox proportional hazards model for fracture risk per SD change in lumbar spine BMD or TBS over time was adjusted for baseline FRAX scores. The statistical analyses were performed using SPSS version 12.0 for Windows (SPSS Inc., Chicago, IL, USA).

## 3. Results

### 3.1. Subject Characteristics

**Figure 1:**
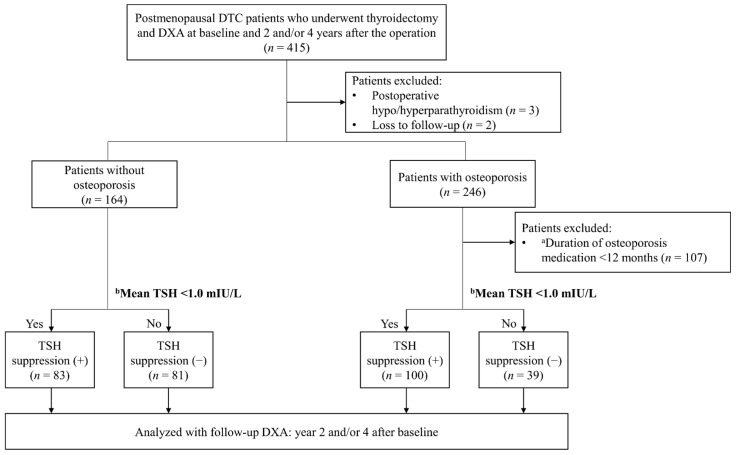
Flowchart of subjects. ^a^ Osteoporosis medication included bisphosphonate, denosumab, selective estrogen receptor modulators (SERMs), and hormone replacement therapy (HRT). ^b^ Mean TSH, the integral value of TSH over time divided by the total follow-up period.

Among 415 postmenopausal DTC patients who had baseline and year 2 and/or year 4 follow-up DXA examinations (Figure 1), 410 patients were enrolled after excluding five patients for the following reasons: postoperative hypoparathyroidism or hyperparathyroidism (*n* = 3) and loss to follow-up (*n* = 2). Among them, 164 patients who had no osteoporosis at the time of surgery were finally enrolled as the ‘patients without osteoporosis’ group. Among the remaining 246 patients who were diagnosed with osteoporosis, 139 patients who received osteoporosis medication for more than 1 year were finally enrolled as the ‘patients with osteoporosis’ group. Next, based on mean TSH 1 mIU/L, patients were further divided into the TSH suppression (−) and the TSH suppression (+) groups.

### 3.2. Changes of BMD and TBS in Patients without Osteoporosis According to TSH Suppression

First, 164 patients without osteoporosis were analyzed. The clinical and biochemical characteristics of the subjects are summarized in Table 1. The TSH suppression (+) group had significantly lower mean TSH levels (2.07 ± 1.15 vs. 0.42 ± 0.32 µIU/mL, *p* = 0.000), a higher proportion of patients who underwent total thyroidectomy (79.0% vs. 92.8%, *p* = 0.011), and a higher total radioactive iodine dose (68.3 ± 20.2 vs. 83.6 ± 36.0 millicurie (mCi), *p* = 0.044) than the TSH suppression (−) group. Other factors, including diabetes mellitus (7.4% vs. 9.6%, *p* = 0.609), cancer type (papillary thyroid carcinoma, 97.5% vs. 98.8%, *p* = 0.546), and cancer stage (stage I, 74.1% vs. 60.2%; stage II, 18.5% vs. 25.3%; stage III, 7.4% vs. 14.5%; *p* = 0.143), were similar in both groups.

Lumbar spine BMD and TBS of each group at baseline, year 2, and year 4 are shown in Table 2. In the TSH suppression (−) group, 70.4% and 64.2% patients underwent DXA at year 2 and year 4, respectively. In the TSH suppression (+) group, 81.9% and 62.7% patients underwent DXA at year 2 and year 4, respectively. The lumbar spine BMD values at baseline, year 2, and year 4 were similar in the TSH suppression (−) group and the TSH suppression (+) group (1.062 ± 0.129 vs. 1.088 ± 0.133, *p* = 0.194). Total hip BMD was also comparable between the two groups at each time point (baseline, 0.917 ± 0.103 vs. 0.925 ± 0.114 g/cm^2^, *p* = 0.635; year 2, 0.895 ± 0.092 vs. 0.896 ± 0.101 g/cm^2^, *p* = 0.943; year 4, 0.892 ± 0.113 vs. 0.895 ± 0.098 g/cm^2^, *p* = 0.899). Although BMD and BMI showed a significant positive correlation (correlation coefficient 0.19, *p* < 0.05), there was no significant correlation between TBS and BMI (correlation coefficient −0.06, *p* = 0.49) (Supplementary Figure S1).

Similar results were found in the TSH suppression (−) and TSH suppression (+) groups for TBS at baseline (1.338 ± 0.071 vs. 1.331 ± 0.075, *p* = 0.532) and year 2 (1.314 ± 0.082 vs. 1.295 ± 0.095, *p* = 0.301), but those at year 4 (1.332 ± 0.081 vs. 1.250 ± 0.131, *p* = 0.000) were significantly higher in the TSH suppression (−) group.

**Figure 2:**
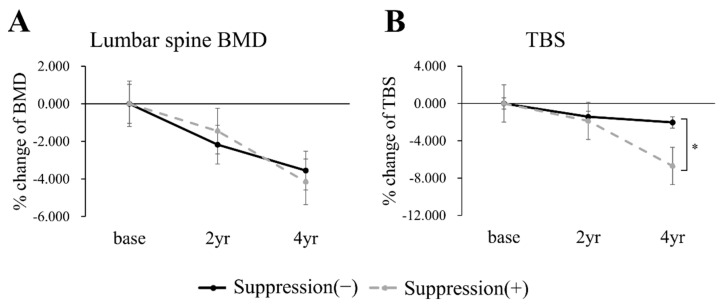
Percentage changes from baseline to years 2 and 4 in lumbar-spine BMD (**A**) and TBS (**B**) in patients without osteoporosis. Black continuous line, the TSH suppression (−) group; gray dotted line, the TSH suppression (+) group. Asterisk bracket (*) indicates *p* value under 0.05.

The percent changes of lumbar spine BMD and TBS are shown in Figure 2. BMD and TBS both steadily decreased in both groups. The linear mixed model showed a significant intergroup difference over time only for TBS (lumbar-spine BMD, *p* = 0.595; total hip BMD, *p* = 0.386; and TBS, *p* = 0.003). The percent change of TBS at year 2 was similar, but the TSH suppression (+) group showed a significantly larger decrease at year 4 than the TSH suppression (−) group.

To further investigate the clinical impacts of TBS scoring, we then explored the fracture assessment. During the median 3 (range of 1–12) year follow-up period, there was no clinical fracture based on the medical records of all patients. Therefore, asymptomatic vertebral fracture was assessed by analyzing T-L-S spine X-ray images and classified according to the Genant classification [22]. A total of 52 patients had lateral spine X-ray; 25 and 27 patients were in the TSH suppression (−) and TSH suppression (+) groups, respectively. The median follow-up duration was not different between groups, i.e., 2 (range of 0.16–10.0) and 3 (range of 0.7–12) years in TSH suppression (−) and TSH suppression (+) groups, respectively. Four patients had fracture with Genant grade 1 in the TSH suppression (−) group, while nine patients (seven patients and two patients had fracture with Genant grade 1 and 2, respectively) had fracture in the TSH suppression (+) group. Interestingly, the Cox proportional hazard model showed that the relative risk of fracture in the TSH suppression (+) group was significantly decreased by 1 SD increase in TBS change (92.4%; 95% CI, 86.1% to 99.2%; *p* < 0.05), but not in BMD change (*p* = 0.628). However, both BMD (*p* = 0.547) and TBS (*p* = 0.843) did not significantly predict fracture risk in the TSH suppression (−) group.

### 3.3. Changes of BMD and TBS in Patients with Osteoporosis According to TSH Suppression

**Figure 3:**
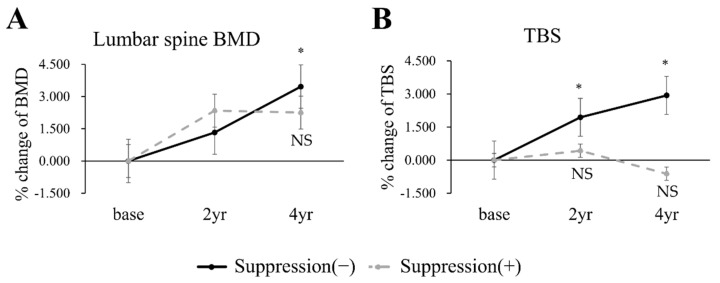
Percentage changes from baseline to years 2 and 4 in lumbar-spine BMD (**A**) and TBS (**B**) in patients with osteoporosis. Black continuous line, the TSH suppression (−) group; gray dotted line, the TSH suppression (+) group. * *p* < 0.05 vs. base in each TSH suppression group. NS indicates ‘not significant’ vs. base in each TSH suppression group.

To further examine the clinical impacts of TBS, then, we analyzed 139 patients with osteoporosis. The baseline demographics are described in Table 3. Age, BMI, diabetes mellitus, and blood test showed no significant difference. Bisphosphonate was the most frequently used medication to treat osteoporosis and 65 (46.8%) patients received more than two types of medication. There was no significant difference in medication types between groups (*p* = 0.523, Table 3). Figure 3 and Supplementary Table S1 demonstrate the changes of BMD and TBS in response to the medical treatment for osteoporosis. At baseline, both BMD and TBS showed no difference between the TSH suppression (−) and the TSH suppression (+) groups (Supplementary Table S1). In the TSH suppression (−) group, both BMD (Figure 3A) and TBS (Figure 3B) were significantly increased at 4 years. However, in the TSH suppression (+) group, both BMD (Figure 3A) and TBS (Figure 3B) showed no significant increase from base to year 2 or 4.

## 4. Discussion

In this study, we compared the change of BMD and TBS over time in postmenopausal DTC patients who underwent surgery. When divided by mean TSH 1.0 mIU/L, there was no substantial difference in BMD at baseline, year 2, and year 4 between the TSH suppression (−) and TSH suppression (+) groups. In contrast, TBS showed a significant intergroup difference at year 4. In a previous study including 84 postmenopausal Caucasian women with DTC, TBS significantly decreased from baseline to 10 years after surgery, whereas BMD did not change [23]. In another study, Moon et al. [21] reported a negative correlation of TBS with the duration of TSH suppression therapy in 273 Korean postmenopausal DTC patients, but a similar correlation was not found for BMD. These results imply that TBS might be more sensitive to TSH suppression therapy than BMD.

BMD and TBS are both indicators of bone strength; BMD represents bone quantity and TBS describes bone quality. Since the BMD is obtained by two-dimensional analysis of DXA, the prediction of bone mass can be complicated by vascular calcifications, vertebral fractures, and osteophytes, which result in exaggeration of BMD [24,25,26]. Due to these limitations, the use of TBS is gradually increasing, and its correlation with bone density has been demonstrated for chronic diseases. For example, type 1 diabetes mellitus patients showed a significantly lower TBS than a healthy population matched for age, sex, and body mass index, while BMD showed no significant difference [27]. Patients with acromegaly showed a higher risk of fracture [28] and a significantly lower TBS [29] than a healthy population, but with no significant difference in BMD. Even in a healthy population, TBS reflects bone density more sensitively than BMD (15–17). In the present study, TBS showed considerable differences between groups, both in absolute values and in percent change. To compare changes over time in the repeated measurements of BMD and TBS, a linear mixed model was used. From baseline to years 2 and 4, the TSH suppression (+) group showed significantly greater deterioration in TBS than the TSH suppression (−) group, but this pattern was not seen for BMD. These results are consistent with previous studies that found no significant correlation between BMD and TSH suppression therapy in DTC patients [30,31].

The relative risk of vertebral fracture, calculated by Cox proportional hazards model, significantly decreased per 1 SD increase in % change of TBS. However, % change of BMD showed no significant prediction of fracture risk. The opposite result was presented by Leslie et al. [32] including 9044 women with age 40 years and older. Because the study population in that study was different from our patients, the difference might imply that TBS is a more advantageous index in postmenopausal DTC patients than in a healthy population.

Since DXA image quality decreases with increasing soft tissue thickness, in the past, the higher the BMI, the lower the TBS [33,34]. However, recent TBS algorithms have no BMI dependency in GE/Lunar scanner by considering this effect in patients with BMI range 15–37 kg/m^2^ [35], as in our study.

Unlike previous studies that analyzed longer-term data, i.e., for 4 years or more and even extending to 10 years and beyond [21], we analyzed BMD and TBS at a shorter-term follow-up of 2 years, as well as at 4 years. By doing so, our data provided insights into whether a short duration of TSH suppression therapy (2 years) was likely to influence the lumbar-spine microstructure. The finding that the TSH suppression (−) and TSH suppression (+) groups showed no significant difference in TBS at 2 years implies that short-term TSH suppression therapy may have a negligible effect on both BMD and TBS.

According to a study by Kim et al., neither TBS nor BMD reflected changes in bone density in postmenopausal DTC patients [20]. In that single-center study, Kim et al. analyzed 4 years of data from 36 patients and found no significant difference in TBS after TSH suppression therapy. Their result was from a group with paired *t*-test, but we used a linear mixed model to compare the intergroup difference depending on whether TSH suppression therapy was performed or not. If they had performed intergroup analysis with postmenopausal DTC patients not on TSH suppression therapy, there might have been a significant difference as there was in our study.

In the analysis with postmenopausal DTC patients taking osteoporosis medication, unlike the prior analysis with patients not taking medication, both BMD and TBS showed an increasing tendency. This result is supposed to originate from administration of bisphosphonate, denosumab, or SERM/HRT during follow-up. However, TBS at year 4 in the TSH suppression (+) group significantly decreased. It seemed that the TSH suppression therapy alleviated the anti-osteoporotic effect of medication.

This study has several limitations. First, the TSH suppression (+) group had a significantly higher rate of thyroidectomy than the TSH suppression (−) group. However, because the cancer stage and tumor type were similar in both groups, the effect of this difference is thought to be minimal, and this difference most likely arose from different protocols regarding the use of surgical methods between the two hospitals. Second, the number of patients in each group gradually decreased over the course of follow-up, and further research is needed in larger cohorts.

## 5. Conclusions

In conclusion, TBS deteriorated more significantly than BMD in postmenopausal DTC patients receiving TSH suppression therapy. A further study with a larger sample size and longer-term follow-up evaluating fracture incidence is needed.

## Figures and Tables

**Table 1:** Baseline characteristics of patients without osteoporosis according to the thyroid-stimulating hormone (TSH) suppression.

	TSH Suppression (−)	TSH Suppression (+)	*p*-Value
*n*	81	83	
Age (years)	56.6 ± 6.6	56.6 ± 6.3	0.986
BMI (kg/m^2^)	23.8 ± 2.7	24.6 ± 3.7	0.141
Cancer type: PTC, *n* (%)	79 (97.5)	82 (98.8)	0.546
TNM stage, *n* (%)			0.143
Stage I	60 (74.1)	50 (60.2)	
Stage II	15 (18.5)	21 (25.3)	
Stage III	6 (7.4)	12 (14.5)	
Extent of surgery: Total thyroidectomy, *n* (%)	64 (79.0)	77 (92.8)	0.011
RAI therapy, *n* (%)	30 (37.0)	48 (42.2)	
Cumulative dose of RAI (mCi)	68.3 ± 20.2	83.6 ± 36.0	0.044
^a^ Mean TSH (mIU/L)	2.07 ± 1.15	0.46 ± 0.32	0.000
**Laboratory test at the time of diagnosis (pre-operative time)**			
TSH (mIU/L)	1.86 ± 1.04	1.97 ± 1.66	0.606
Free T4 (ng/dL)	1.20 ± 0.23	1.17 ± 0.31	0.523
Calcium (mg/dL)	9.25 ± 0.41	9.14 ± 0.42	0.113
Phosphate (mg/dL)	3.81 ± 0.55	3.73 ± 0.46	0.300
ALP (IU/L)	62.80 ± 15.90	66.86 ± 22.81	0.190
25OHD (ng/mL)	18.06 ± 9.37	18.54 ± 9.69	0.829
iPTH (pg/mL)	35.34 ± 20.95	39.68 ± 28.23	0.298

^a^ Mean TSH, the integral value of TSH over time divided by the total follow-up period. Values are presented as mean ± standard deviation for continuous variables. Comparisons between the two groups were made using the parametric Student *t*-test. Abbreviations: PTC, papillary thyroid carcinoma; RAI, radioactive iodine; BMI, body mass index; 25OHD, 25-hydroxy vitamin D3; iPTH, intact PTH; ALP, alkaline phosphatase.

**Table 2:** Changes of bone mineral density (BMD) and trabecular bone score (TBS) in patients without osteoporosis.

	Suppression (−)	Suppression (+)	*p*-Value
L-BMD (g/cm^2^)			
Baseline	1.062 ± 0.129	1.088 ± 0.133	0.194
Year 2	1.031 ± 0.136	1.053 ± 0.113	0.308
Year 4	1.036 ± 0.090	1.033 ± 0.114	0.903
TBS			
Baseline	1.338 ± 0.071	1.331 ± 0.075	0.532
Year 2	1.314 ± 0.082	1.295 ± 0.095	0.301
Year 4	1.332 ± 0.081	1.250 ± 0.131	0.000

Values are presented as mean ± standard deviation for continuous variables. Comparisons between the two groups at baseline, year 2, and year 4 were made using the Student *t*-test. Abbreviations: L-BMD, bone mineral density of the lumbar spine; TBS, trabecular bone score.

**Table 3:** Baseline characteristics of patients with osteoporosis according to the TSH suppression.

	TSH Suppression (−)	TSH Suppression (+)	*p*-Value
*N*	100	39	
Age (years)	61.0 ± 8.0	60.0 ± 8.0	0.857
BMI (kg/m^2^)	23.6 ± 3.1	24.6 ± 4.3	0.126
Diabetes mellitus, *n* (%)	25 (25.0)	8 (20.5)	0.576
^a^ Medications for osteoporosis			0.523
Bisphosphonate	89 (89.0)	34 (87.2)	
Denosumab	24 (24.0)	8 (20.5)	
SERM/HRT	50 (50.0)	14 (35.9)	
Cancer type: PTC, *n* (%)	94 (94.0)	34 (87.2)	0.181
Extent of surgery: Total thyroidectomy, *n* (%)	97 (89.9)	28 (71.8)	<0.001
^b^ Mean TSH (mIU/L)	2.51 ± 1.43	0.32 ± 0.25	<0.001
**Laboratory test at the time of diagnosis (pre-operative time)**			
TSH (mIU/L)	2.15 ± 4.57	2.95 ± 4.02	0.313
Free T4 (ng/dL)	1.32 ± 0.18	1.24 ± 0.21	0.050
Calcium (mg/dL)	9.00 ± 0.66	8.92 ± 0.43	0.434
Phosphate (mg/dL)	3.78 ± 0.64	3.83 ± 0.53	0.657
ALP (IU/L)	60.96 ± 19.82	63.97 ± 30.25	0.567
25OHD (ng/mL)	25.11 ± 18.84	20.83 ± 14.43	0.193
iPTH (pg/mL)	23.12 ± 14.88	21.59 ± 12.56	0.587

^a^ Medications for osteoporosis, patients who received more than one type of medication were counted by each type of medication. ^b^ Mean TSH, the integral value of TSH over time divided by the total follow-up period. Values are presented as mean ± standard deviation for continuous variables. Comparisons between the two groups were made using the parametric Student *t*-test. Abbreviations: BMI, body mass index; PTC, papillary thyroid carcinoma; SERM, selective estrogen receptor modulator; HRT, hormone replacement therapy; 25OHD, 25-hydroxy vitamin D3; iPTH, intact PTH; ALP, alkaline phosphatase.

## Data Availability

The data presented in this study are available on request from the corresponding author. The data are not publicly available due to privacy issue.

## Supplementary Materials

The following are available online at https://www.mdpi.com/article/10.3390/jcm10091964/s1, Figure S1: Correlation analysis between BMI and BMD (A), and between BMI and TBS (B) in postmenopausal DTC patients without osteoporosis, Table S1: Changes of BMD and TBS in patients with osteoporosis.
