# Supplementary material for: Measurements of Bone Health after Thyroid-Stimulating Suppression Therapy in Postmenopausal Women with Differentiated Thyroid Carcinoma: Bone Mineral Density versus the Trabecular Bone Score
Source: J Clin Med. 2021 May 3;10(9):1964. doi: 10.3390/jcm10091964 (PMC8125563; doi:10.3390/jcm10091964)
Supplement: Supplementary file 1 [file jcm-10-01964-s001.zip › jcm-1198950-supplementary.pdf]

Supplementary Table & Figure

Supplementary Table 1. Changes of BMD and TBS in patients with osteoporosis

	**Suppression (–)**	**Suppression (+)**	***p*-value**
L-BMD (g/cm^2)			
Baseline	0.878 ± 0.155	0.889 ±0.148	0.705
Year 2	0.880 ±0.140	0.890 ± 0.108	0.68
Year 4	0.898 ± 0.134	0.901 ± 0.124	0.920
TBS			
Baseline	1.309 ±0.079	1.303 ± 0.079	0.668
Year 2	1.330 ± 0.084	1.310 ± 0.009	0.241
Year 4	1.338 ±0.072	1.296 ± 0.089	0.012

Values are presented as mean ± standard deviation for continuous variables. Comparisons between the two groups at baseline, year 2, and year 4 were made using the Student t-test. Abbreviations: L-BMD, bone mineral density of the lumbar spine; TBS, trabecular bone score.

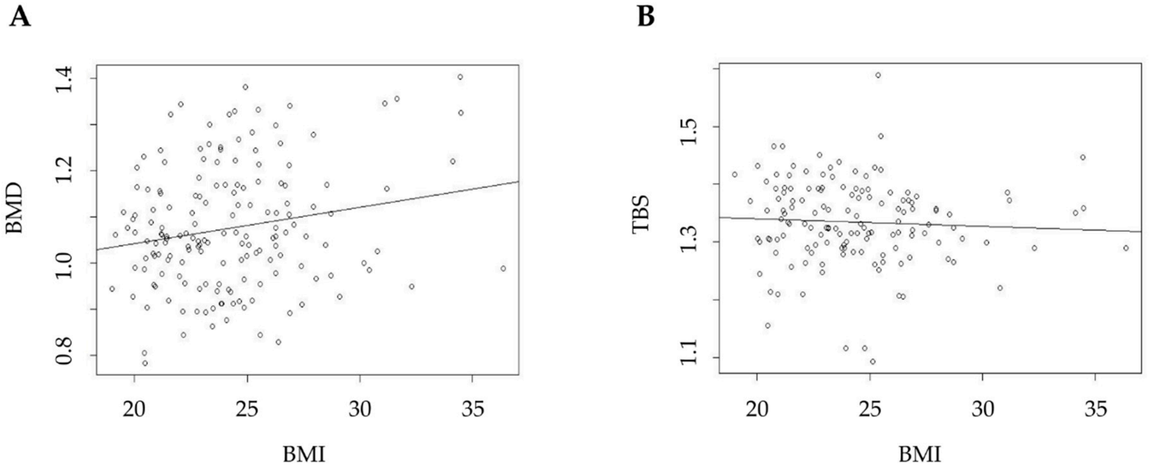

Supplementary Figure 1. Correlation analysis between BMI and BMD (**A**), and between BMI and TBS (**B**) in postmenopausal DTC patients without osteoporosis.
